# Impact of the COVID-19 Pandemic on COPD Patient Mortality: A Nationwide Study in France

**DOI:** 10.3389/ijph.2024.1606617

**Published:** 2024-02-01

**Authors:** Jonas Poucineau, Myriam Khlat, Nathanaël Lapidus, Maude Espagnacq, Christos Chouaïd, Tristan Delory, Sophie Le Coeur

**Affiliations:** ^1^ Institut National d’Études Démographiques (INED), Paris, France; ^2^ Institut de Recherche et Documentation en Économie de la Santé (IRDES), Paris, France; ^3^ Faculté de Santé, Sorbonne Université, Paris, France; ^4^ Institut National de la Santé et de la Recherche Médicale (INSERM) U1136 Institut Pierre Louis d’Epidémiologie et de Santé Publique, Paris, France; ^5^ Hôpital Saint-Antoine, Paris, France; ^6^ Institut National de la Santé et de la Recherche Médicale (INSERM) U955 Institut Mondor de Recherche Biomédicale (IMRB), Créteil, France; ^7^ Hospital Center Intercommunal De Créteil, Créteil, France; ^8^ Centre Hospitalier Annecy Genevois (CH Annecy), Metz-Tessy, France

**Keywords:** COPD, COVID-19 pandemic, impacts, mortality, population-based study

## Abstract

**Objectives:** We investigated the mortality patterns of chronic obstructive pulmonary disease (COPD) patients in France relative to a control population, comparing year 2020 to pre-pandemic years 2017–2019.

**Methods:** COPD patient and sex, age and residence matched control cohorts were created from the French National Health Data System. Survival was analyzed using Cox regressions and standardized rates.

**Results:** All-cause mortality increased in 2020 compared to 2019 in the COPD population (+4%), but to a lesser extent than in the control population (+10%). Non-COVID-19 mortality decreased to a greater extent in COPD patients (−5%) than in the controls (−2%). Death rate from COVID-19 was twice as high in the COPD population relative to the control population (547 vs. 279 per 100,000 person-years).

**Conclusion:** The direct impact of the pandemic in terms of deaths from COVID-19 was much greater in the COPD population than in the control population. However, the larger decline in non-COVID-19 mortality in COPD patients could reflect a specific protective effect of the containment measures on this population, counterbalancing the direct impact they had been experiencing.

## Introduction

Patients with chronic obstructive pulmonary disease (COPD) are a vulnerable population, due to their relatively old age and the comorbidities frequently associated with the disease. When infected by SARS-CoV-2, they were shown to have worse outcomes and a higher risk of mortality than the rest of the population [1–5]. This was particularly observed among severe COPD patients [6]. Therefore, COPD patients may undergo a greater direct mortality impact from the pandemic. However, the balance of the direct and indirect impacts of the pandemic on those patients is hard to demonstrate, as several factors may have come into play, with counter-balancing effects.

First, a harvesting effect may have occurred, i.e., an excess mortality due to an exogenous shock anticipating the death of the frailest categories of the population, compensated by a decrease in mortality in the following weeks/months [7–9]. If such an effect occurred during the COVID-19 pandemic, all-cause mortality is expected to increase during pandemic waves, and decrease subsequently in the following months. Second, the reorganization of hospital care for management of symptomatic COVID-19 patients, and hospital avoidance behaviors for fear of becoming infected with COVID-19 [10–12] may have led to disruptions in medical care, with possible negative consequences on the health of patients with chronic diseases [13, 14]. In that case, all-cause mortality is expected to increase during pandemic waves and possibly in the following months. Third, the sanitary measures implemented, including lockdowns, the closure of social venues and face mask use and social distancing instructions, may have had a protective effect on COPD patients’ health, in particular by decreasing the circulation of respiratory viruses, involving a reduction in acute exacerbations of COPD (AECOPD) [15–18]. In that case, non-COVID-19 mortality is expected to decrease during pandemic waves, particularly for deaths associated with respiratory causes.

To date, a significant decrease in hospital admissions for acute exacerbations of COPD (AECOPD) has been observed worldwide [12, 15–22], and the rare studies on mortality showed a positive effect of the pandemic on the survival of COPD patients. In Slovenia, COPD patient all-cause mortality was reported to decrease slightly in 2020 compared to pre-pandemic years 2016–2019 (−4%) [21], while in Denmark a sharp decrease has been observed during the first half of 2020 compared to the first half of 2019 (−17%) [18]. Those preliminary findings have yet to be confirmed by large population-based studies of COPD patient mortality, with appropriate control populations to assess changes in differential mortality. More generally, the net impact of the pandemic on COPD patient mortality remains to be explored, especially in a context of high pandemic intensity. France is of potential interest for this purpose, as it was one of the most hardly hit European countries, particularly during the first wave [23]. The country also stands out with respect to the health measures implemented, rated as particularly stringent by the Oxford COVID-19 Government Response Tracker [24]. These measures were assessed as globally effective during the first wave by a study comparing COVID-19 containment measures in several European countries, even though the country could have benefited from an extension of the lockdown period [25].

Based on exhaustive health data at the national level, the present study describes the mortality patterns of COPD patients in France relative to a control population, comparing year 2020 to pre-pandemic years 2017–2019, in order to analyze the direct and indirect impacts of the pandemic. The specific objectives of the study were to assess in COPD and control cohorts: 1) the levels of total mortality and mortality from non-COVID-19 causes in 2020 in comparison with the pre-pandemic situation and; 2) the levels of COVID-19 mortality in 2020. Mortality gaps were analyzed in different ways in order to provide a complete picture of the effects of the pandemic.

## Methods

This observational study was conducted according to STROBE guidelines [26].

### Database: National Health Data System

The study was based on the National Health Data System (*Système National des Données de Santé,* SNDS), an exhaustive and nationwide administrative health claims database, covering 99% of the French population, i.e., over 66 million people [27]. The SNDS encompasses 1) all prescription-based medication deliveries with corresponding Anatomical Therapeutic Chemical (ATC) codes and dates of delivery, 2) all hospitalizations in the public and private sector with corresponding International Classification of Diseases 10th Revision (ICD10) codes for primary and associated diagnoses, and dates of admission and discharge, and 3) a repository listing the payment exemptions for people with long-term condition(s) (LTC) with corresponding ICD10 codes. The database also contains demographic data, including sex and dates of birth and death. Causes of death are also available for the year 2020.

No missing data had to be processed, as all variables of interest were correctly filled in.

### Design of the Study

The study period extended from January 2017 to December 2020 and was divided into two main periods: the pre-pandemic years 2017–2019 and the pandemic year 2020. The first two pandemic wave periods were defined based on a threshold of 100 COVID-19 deaths per day at the national level. The year 2020 has thus been subdivided into four sub-periods, compared to the same sub-periods of the pre-pandemic years: 1 January to 16 March, 17 March to 22 May (first pandemic wave), 23 May to 11 October; and 12 October to 31 December (second pandemic wave). The 2nd and 3rd sub-periods were specifically investigated to analyze the direct and indirect effects of the first COVID-19 wave.

Using an algorithm adapted from the French National Authority for Health [28], four cohorts of COPD patients were set up, identified on 1 January of each year from 2017 to 2020 and followed up until 31 December of the corresponding year, i.e., for an entire year. Patients were identified based on their healthcare use in the years prior to inclusion in the cohort. Therefore, there was no overlap between the identification and follow-up periods: patients identified on 1 January 2017 based on their healthcare use in previous years were followed until 31 December 2017; those identified on 1 January 2018 were followed until 31 December 2018, etc. As the study was based on prevalent patients, individuals could be included in more than one cohort, generating homogeneity within the population. This has been taken into account using a cluster option providing a robust variance in the analyses.

For each COPD cohort, a corresponding control cohort (individuals without COPD) was created from a stratified random sample, with a ratio of two controls per case, matched on sex, age and area of residence. As for COPD cohorts, control cohorts were formed on 1 January of each year from 2017 to 2020, and followed up until 31 December of the corresponding year.

### Identification of COPD Patients

**FIGURE 1:**
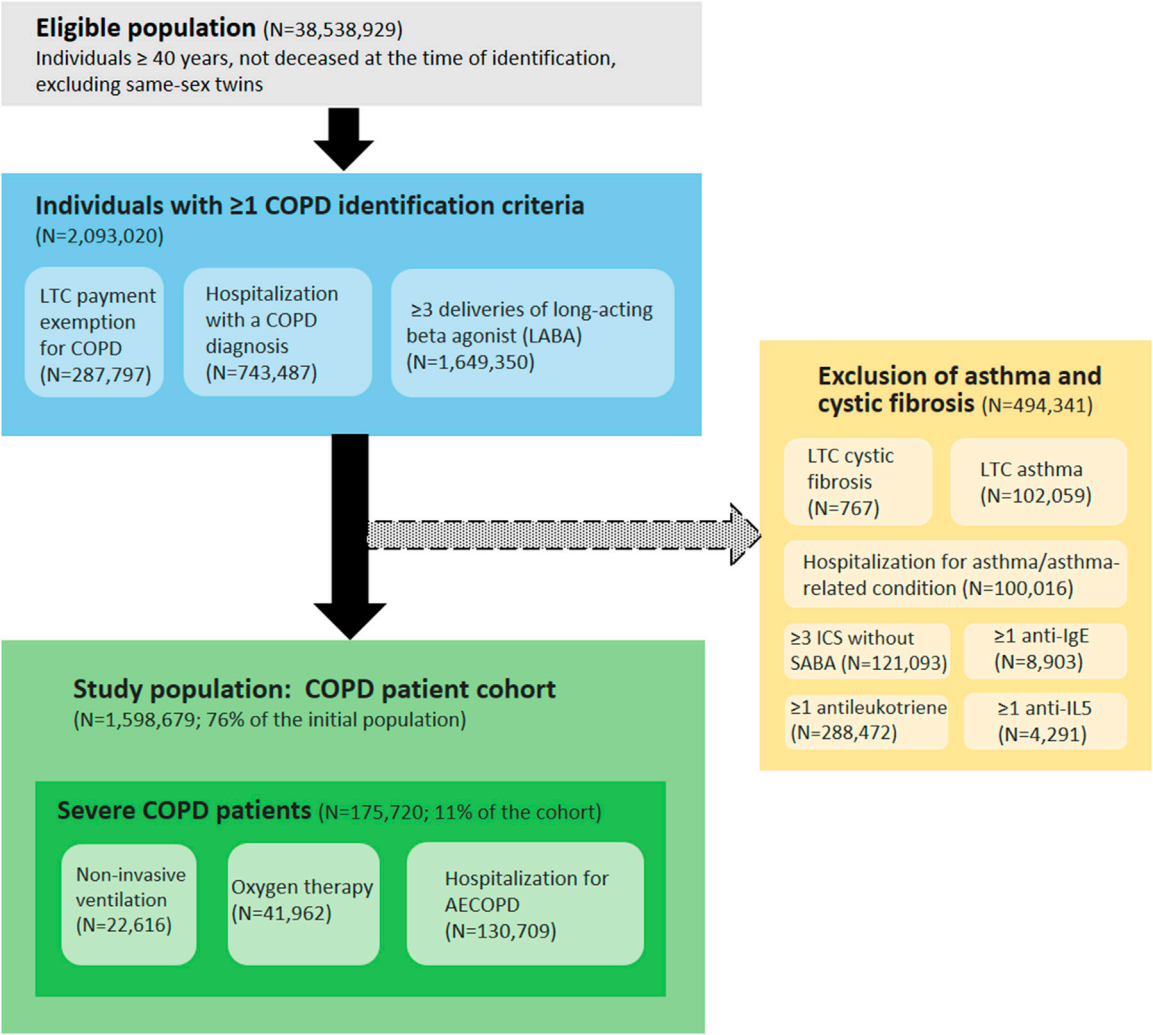
Algorithm for identifying patients with COPD based on healthcare use in the National Health Dada System (France, 2020 cohort). Abbreviations: COPD, chronic obstructive pulmonary disease; LTC, long-term condition; ICS, inhaled corticosteroids; SABA, short-acting beta agonist; anti-IgE, anti-immunoglobulin E; anti-IL5, anti-interleukin-5; AECOPD, acute exacerbation of COPD.

The COPD patient identification algorithm included individuals aged 40 years or older, with at least one of the inclusion criteria and no exclusion criterion (presence of asthma or cystic fibrosis) (Figure 1).

Inclusion criteria:

- Payment exemption for an LTC (ICD10 code J41, J42, J43, J44 or J96.1)
- Hospitalization with COPD as a primary or associated diagnosis in the last 5 years (ICD10 code J41, J42, J43, J44 or J96.1)
- At least three deliveries of long-acting beta agonist (LABA) in the last year (ATC code R03AC13, R03AC18, R03AC19, R03AC12, R03BB06, R03BB04, R03BB07, R03AL04, R03AL06, R03BB54, R03AL03, R03AK08, R03AK06, R03AK07, R03AK10, R03AL09 or R03AL08)

Exclusion criteria:

- Payment exemption for an LTC for asthma (ICD10 code J45) or cystic fibrosis (ICD10 code E84)
- Hospitalization with asthma or asthma-related condition as a primary or associated diagnosis in the last 2 years (ICD10 code J45 or J46)
- At least three deliveries of inhaled corticosteroids (ICS) (ATC code R03BA01, R03BA02 or R03BA05) without same-day delivery of short-acting beta agonist (SABA) (ATC code R03AC02, R03AC03, R03BB01 or R03AL01) in the last 2 years
- At least one delivery of anti-immunoglobulin E (anti-IgE) in the last 2 years (ATC code R03DX05)
- At least one delivery of anti-interleukin-5 (anti-IL5) in the last 2 years (ATC code R03DX09 or R03DX08)
- At least one delivery of antileukotriene in the last 2 years (ATC code R03DC03)

In addition, within the COPD cohorts, patients with at least one of the following criteria were considered as severe COPD patients:

- Oxygen therapy at home in the last year, identified by medical act codes and medical device codes in the SNDS
- Non-invasive ventilation in the last year, identified by medical act codes and medical device codes in the SNDS
- Hospitalization for AECOPD in the last 5 years, identified from the primary diagnoses (PD) and associated diagnoses (AD) with compatible ICD10 codes: J44.0 or J44.1 as PD; or J96.0 as PD and (J43 or J44) as AD; or (J09-18 or J20-22) as PD and (J43 or J44) as AD; or (J43 or J44) as PD and (J44.0 or J44.1 or J96.0 or J09-18 or J20-22) as AD. Stays lasting fewer than 2 days were excluded, in order to eliminate scheduled hospitalizations for check-up examinations [29].

### Statistical Analyses

The main outcomes of this study were all-cause mortality, COVID-19 mortality and non-COVID-19 mortality. COVID-19 mortality corresponds to deaths due to COVID-19 as the underlying cause, and non-COVID-19 mortality corresponds to deaths due to any other underlying cause. Survival analyses were computed using Cox regressions to model the risk of death, adjusting for sex and age groups (40–49 years, 50–59, 60–69, 70–79, and 80 or older). Intra-group models were computed to estimate the variations between two cohorts in a given population (COPD or control). All-cause and non-COVID-19 mortality were compared between year 2020 and year 2019. Inter-group models were also computed to estimate the variations between the COPD and control populations in a given year (2017, 2018, 2019 and 2020). Interactions between population (COPD or control) and period of follow-up were tested to assess the significance of variations between models. Age and sex standardized mortality rates were computed (per 100,000 person-years) for all-cause, COVID-19 and non-COVID-19 deaths, with the average COPD population in the pre-pandemic years 2017–2019 as the reference population. Year 2020 was compared with pre-pandemic years 2017–2019 to analyze absolute variations in mortality.

## Results

### Description of the Populations

COPD cohorts included 1,432,653, 1,475,323, 1,522,101 and 1,598,679 individuals, respectively, among whom 79,440, 83,159, 83,326 and 90,765 deaths were observed in 2017, 2018, 2019 and 2020, respectively. Control cohorts included 2,865,306, 2,950,646, 3,044,202 and 3,197,358 individuals, respectively, among whom 67,108, 70,996, 71,069 and 81,069 deaths were observed in 2017, 2018, 2019 and 2020, respectively. Overall, the median age was 68 years [interquartile range (IQR): 59–78], and 56% of individuals were men. Each COPD cohort was composed of 11% severe patients and 89% non-severe patients. Severe COPD patients were older than non-severe patients (median: 74 years, IQR: 65–83 in severe patients; median: 68, IQR: 58–78 in non-severe patients).

### All-Cause Mortality in the Pre-Pandemic Period

**FIGURE 2:**
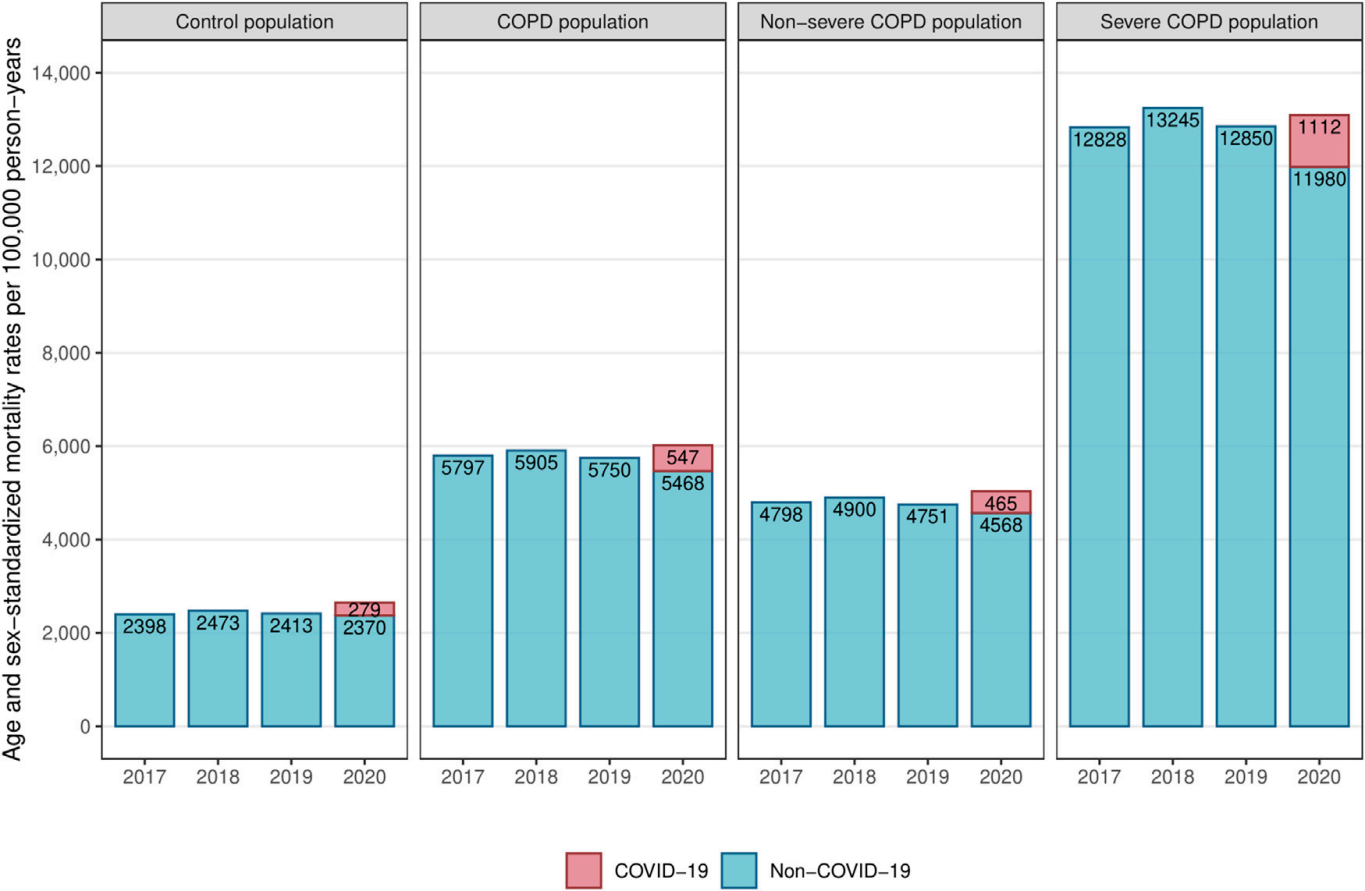
Yearly age-sex-standardized mortality rates in the control, COPD, severe COPD and non-severe COPD populations (France, 2017–2020). Abbreviation: COPD, chronic obstructive pulmonary disease.

In the pre-pandemic period (years 2017–2019), age-sex-standardized all-cause mortality rates were relatively stable within each population, as the annual variations did not exceed 3% (Figure 2). Among the COPD population, all-cause mortality rate was more than twice as high as in the control population (5,800 vs. 2,400 deaths per 100,000 person-years). Severe COPD patients had an all-cause mortality rate more than five times higher than the control population (13,000 vs. 2,400 deaths per 100,000 person-years).

### All-Cause Mortality: Pre-Pandemic vs. Pandemic Periods

In 2020, standardized all-cause mortality rates increased in both the COPD and control populations compared to pre-pandemic averages (Figure 2; Table 1). The COPD population experienced a slightly lower increase than the control population (+198 vs. +221 excess deaths per 100,000 person-years). Within the COPD population, severe patients had a smaller increase than non-severe patients (+123 vs. +217 excess deaths per 100,000 person-years).

**TABLE 1:** Intra-group variations in all-cause, COVID-19 and non-COVID-19 mortality among the control, COPD, severe COPD and non-severe COPD populations (France, 2017–2020).

	All-cause mortality rate^a^			COVID-19 mortality rate in 2020^a^	Non-COVID-19 mortality rate^a^		All-cause mortality risk in 2020 relative to 2019^b^ HR [95% CI]	Non-COVID-19 mortality risk in 2020 relative to 2019^b^ HR [95% CI]
	2017–2019 average	2020	Difference (2020–2017/2019)		2020	Difference (2020–2017/2019)		
Control population	2,428	2,649	+221	279	2,370	−58	1.10 [1.08; 1.11]**	0.98 [0.97; 0.99]**
COPD population	5,817	6,015	+198	547	5,468	−349	1.04 [1.03; 1.05]**	0.95 [0.94; 0.96]**
Non-severe COPD population	4,816	5,033	+217	465	4,568	−247	1.05 [1.04; 1.07]**	0.96 [0.95; 0.97]**
Severe COPD population	12,97	13,093	123	1,112	11,98	−989	1.02 [1.01; 1.04]**	0.93 [0.92; 0.95]**

Abbreviations: COPD, chronic obstructive pulmonary disease; HR, hazard ratio; CI, confidence interval.

Significance levels: *p < 0.05, **p < 0.01.

^a^ Age-sex-standardized rates per 100,000 person years.

^b^ Adjusted for sex, age groups and period.

Survival analyses showed that the overall risk of death from all causes was higher in 2020 compared to 2019 in both the COPD and control populations, but to a lesser extent for the COPD population (hazard ratio (HR) [95% confidence interval]: 1.04 [1.03–1.05]) than for the control population (HR: 1.10 [1.08–1.11]) as confirmed by the interaction test (*p* < 0.01). Within the COPD population, severe patients had a lower increase in all-cause death risk (HR: 1.02 [1.01–1.04]) than non-severe patients (HR: 1.05 [1.04–1.07]).

**FIGURE 3:**
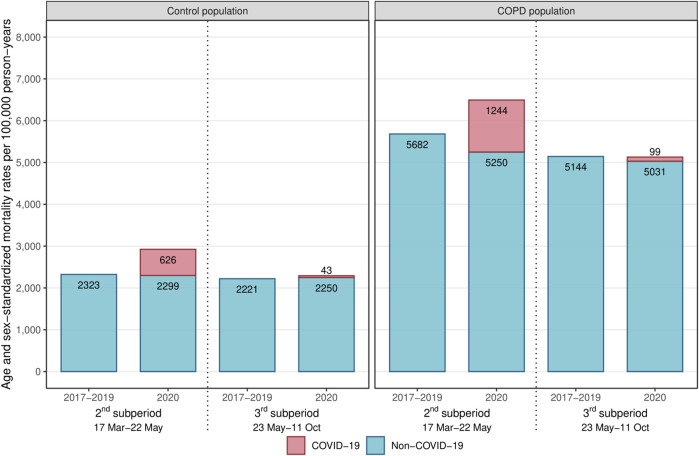
Age-sex-standardized mortality rates during the 2nd and 3rd sub-periods among the control and COPD populations (France, 2017–2020).Abbreviation: COPD, chronic obstructive pulmonary disease.

During the first pandemic wave (2nd sub-period), both the COPD and control populations experienced a dramatic increase in all-cause mortality compared to 2019 (HR: 1.16 [1.14–1.19] and 1.26 [1.23–1.29], respectively) (Figure 3; Table 2). Between the first and second waves (3rd sub-period), all-cause mortality increased only in the control population (HR: 1.03 [1.02–1.05], interaction test: *p* < 0.01).

**TABLE 2:** Intra-group variations in all-cause, COVID-19 and non-COVID-19 mortality during and after the first wave among the control and COPD populations (France, 2017–2020).

	All-cause mortality rate^a^			COVID-19 mortality rate in 2020^a^	Non-COVID-19 mortality rate^a^		All-cause mortality risk in 2020 relative to 2019^b^ HR [95% CI]	Non-COVID-19 mortality risk in 2020 relative to 2019^b^ HR [95% CI]
	2017–2019 average	2020	Difference (2020–2017/2019)		2020	Difference (2020–2017/2019)		
Control population								
1st wave (17 March–22 May)	2,323	2,924	+601	626	2,299	−24	1.26 [1.23; 1.29]**	0.99 [0.96; 1.01]
Between 1st and 2nd wave (23 May–11 October)	2,221	2,293	+72	43	2,250	+29	1.03 [1.02; 1.05]**	1.01 [1.00; 1.03]
COPD population								
1st wave (17 March–22 May)	5,682	6,493	+811	1,244	5,250	−432	1.16 [1.14; 1.19]**	0.94 [0.92; 0.96]**
Between 1st and 2nd wave (23 May–11 October)	5,144	5,130	−14	99	5,031	−113	1.00 [0.98; 1.02]	0.98 [0.97; 1.00]*

Abbreviations: COPD, chronic obstructive pulmonary disease; HR, hazard ratio; CI, confidence interval.

Significance levels: *p < 0.05, **p < 0.01.

^a^ Age-sex-standardized rates per 100,000 person years.

^b^ Adjusted for sex, age groups and period.

### COVID-19 Mortality in 2020

In 2020, COVID-19 mortality was twice as high in the COPD population as in the control population (547 vs. 279 deaths per 100,000 person-years, HR: 2.00 [1.94–2.06]) (Table 1; Table 3). Among severe COPD patients, COVID-19 mortality rate was nearly four times higher than in the control population (1,112 vs. 279 deaths per 100,000 person-years, HR: 3.86 [3.68–4.04]).

**TABLE 3:** All-cause and COVID-19 mortality gap between the control population and the COPD, severe COPD and non-severe COPD populations (France, 2017–2020).

	2017 all-cause mortality^a^	2018 all-cause mortality^a^	2019 all-cause mortality^a^	2020	
				All-cause mortality^a^	COVID-19 mortality^a^
Control population	1 (reference)	1 (reference)	1 (reference)	1 (reference)	1 (reference)
COPD population	2.43 [2.41; 2.46]**	2.41 [2.38; 2.43]**	2.41 [2.38; 2.43]**	2.30 [2.28; 2.32]**	2.00 [1.94; 2.06]**
Non-severe COPD population	2.02 [2.00; 2.04]**	2.00 [1.98; 2.02]**	1.99 [1.97; 2.01]**	1.93 [1.91; 1.95]**	1.69 [1.63; 1.75]**
Severe COPD population	4.91 [4.84; 4.99]**	4.95 [4.88; 5.03]**	4.96 [4.89; 5.03]**	4.66 [4.59; 4.73]**	3.86 [3.68; 4.04]**

Abbreviations: COPD, chronic obstructive pulmonary disease; HR, hazard ratio; CI, confidence interval.

Significance levels: *p < 0.05, **p < 0.01.

^a^ Adjusted for sex, age groups and population (reference = control population).

### Non-COVID-19 Mortality: Pre-Pandemic vs. Pandemic Periods

In 2020, standardized non-COVID-19 mortality rates decreased in each population compared to pre-pandemic averages (Table 1). The COPD population experienced a substantially higher decrease than the control population (−349 vs. −58 deaths per 100,000 person-years). Within the COPD population, severe patients had a greater decrease than non-severe patients (−989 vs. −247 deaths per 100,000 person-years).

Survival analyses showed that the risk of death from causes other than COVID-19 decreased in 2020 compared to 2019 in both the COPD and control populations, but to a greater extent for the COPD population (HR: 0.95 [0.94–0.96]) than for the control population (HR: 0.98 [0.97–0.99], interaction test: *p* < 0.01). Within the COPD population, severe patients had a greater decrease in non-COVID-19 death risk (HR: 0.93 [0.92–0.95]) than non-severe patients (HR: 0.96 [0.95–0.97], interaction test: *p* < 0.01).

In the COPD population, a substantial decrease in non-COVID-19 mortality was observed during the first pandemic wave (2nd sub-period) (HR: 0.94 [0.92–0.96]), and extent smaller decline was observed between the first and second waves (3rd sub-period) (HR: 0.98 [0.97–1.00]), while in the control population, non-COVID-19 mortality risks remained at the same level in 2020 as in 2019 during both sub-periods (Figure 3; Table 2).

### Differential All-Cause Mortality (2017–2020)

Relative to the control population, the COPD population had a +143% higher all-cause risk of death in 2017 (HR: 2.43 [2.41–2.46]), +141% in 2018 (HR: 2.41 [2.38–2.43]), and +141% in 2019 (HR: 2.41 [2.38–2.43]) (Table 3). In 2020, the gap between COPD and control populations narrowed to +130% (HR: 2.30 [2.28–2.32], interaction test: *p* < 0.01). The convergence of all-cause mortality levels with the control population in 2020 was observed in both severe and non-severe COPD patients.

## Discussion

The present study is, to our knowledge, the largest population-based study of the mortality of COPD patients in the context of the COVID-19 pandemic. Based on exhaustive national patient cohorts, the main results of this study are 1) an increase in all-cause mortality in 2020 compared to pre-pandemic years in COPD patients, but to a lesser extent than in the control population, resulting in a reduction in the mortality gap between the two populations, 2) a much higher risk of death from COVID-19 in 2020 for COPD patients compared to the control population and 3) a greater reduction in non-COVID-19 mortality in COPD patients compared to the control population.

While the impact of the pandemic on hospital admissions for COPD has been extensively studied worldwide, the evolution of mortality in COPD patients at population level has been little investigated so far. A cohort study in Slovenia over the period 2016–2020 with a population of 14,000 to 17,000 COPD patients per year showed a slight decrease in all-cause mortality in 2020 compared to previous years (−4%) [21]. Another study in Denmark on a cohort of 17,000 COPD patients showed a decrease in all-cause mortality in the first half of 2020 compared to the first half of 2019 (−17%) [18]. In contrast, our study based on COPD cohorts encompassing 1.5 million patients per year shows that all-cause mortality increased in 2020 compared to 2019 (+4%). However, the control population composed of individuals without COPD with the same age and sex structure experienced a greater increase in all-cause mortality (+10%). Consequently, the all-cause mortality gap between the COPD and control populations narrowed by 11–13 percentage points in 2020 compared to previous years.

Secondly, this study shows that COVID-19 mortality was twice as high in the COPD population compared to the control population, both in terms of standardized rate and risk of death. Several studies in the literature have shown a higher risk of severity or mortality in case of COVID-19 infection for COPD patients compared to the general population [1, 2, 4]. However, the direct mortality impact also depends on the infection rate, which is not known for COPD patients. The population-based approach we have implemented provides more complete information by assessing COVID-19 mortality in COPD patients compared with a control population.

Furthermore, in both the COPD and control populations, COVID-19 mortality rates exceeded the all-cause excess deaths observed in 2020 compared to pre-pandemic years. Accordingly, mortality from causes other than COVID-19 decreased during the first year of the pandemic, and to a larger extent for the COPD population (−5%) than for the control population (−2%). This finding was reinforced for severe COPD patients, who experienced a greater decline in non-COVID-19 mortality in 2020 than non-severe patients compared with previous years (−7% vs. −4%). In Slovenia, Sarc et al. found a greater decrease in non-COVID-19 mortality in 2020 compared to previous years among COPD patients (−15%) [21]. In France, analysis of causes of death at national level has revealed that non-COVID-19 mortality decreased overall in 2020, and this pattern has been evidenced again in 2021. The decline mainly concerned deaths from diseases of the respiratory system, diseases of the nervous system and sense organs, and tumors [30, 31]. In contrast, studies in the United States observed a rise in non-COVID-19 mortality in the general population during the pandemic, in particular for circulatory diseases, diabetes, external causes and drug- or alcohol-induced causes [32–35].

Two main factors may have contributed to the decline in non-COVID-19 mortality. First, a harvesting effect may have occurred. As reported by Cerqua et al., there is little evidence in the literature of a potential harvesting effect associated with the COVID-19 pandemic, except in some Italian regions at the end of the first wave [7, 36, 37]. In a French study on nursing home residents, no mortality displacement effect was observed up until August 2020 [38]. In our study, we found that the COPD population experienced a sharp rise in all-cause mortality during the first wave compared to the same period in 2019 (+16%, see Table 2), with an important proportion of deaths associated with COVID-19, followed by a slight drop in non-COVID-19 mortality in the period between the first and second waves (−2%), reflecting a possible harvesting effect. When focusing on the most affected regions (Ile-de-France, Grand Est and Bourgogne-Franche-Comté) the phenomenon was amplified, with a greater rise in all-cause mortality during the first wave (+45%, data not shown), followed by a marked drop in non-COVID-19 mortality in the following period (−5%). This hypothesis is supported by the fact that such a phenomenon was not observed in the control population. However, if there was a harvesting effect in the COPD population, it was only partial, as the excess all-cause mortality observed in the first wave was much greater than the modest drop in non-COVID-19 mortality in the subsequent period.

The second factor is a potential protective effect of the containment measures implemented in 2020 on COPD patients’ health. Previous studies in India, Hong Kong, Singapore, the United States and Denmark showed that the policy responses to the first COVID-19 wave are likely to have contributed to a reduction in the incidence of severe AECOPD [15–18, 39], which could result in a decline in COPD patient mortality. The results of the present study support this hypothesis, as during the first pandemic wave, non-COVID-19 mortality fell significantly in the COPD population (−6%), while it remained stable in the control population.

Finally, possible interruption or delay of care due to hospital overcrowding and avoidance of care for fear of COVID-19 infection may have had a negative effect on the survival of COPD patients. In our study, non-COVID-19 mortality did not rise during the first pandemic wave in the COPD population, and the increase in all-cause mortality appears to be directly related to COVID-19 deaths. Nonetheless, it is not impossible that such an effect occurred but was compensated by a potential protective effect of sanitary measures and/or a possible harvesting effect.

This study has several limitations. The results highlight several factors that potentially impacted on COPD patient mortality during the pandemic. However, it is not possible to validate these hypotheses with certainty, as it would require more detailed information on prevention measures and hospital saturation at local level. It is not possible either to dissociate the relative weight of each of these factors, as they have opposing effects. Besides, as the database does not contain clinical data at the individual level, we were not able to validate the algorithm for identifying COPD patients. However, we drew on previously published algorithms and worked in consultation with COPD and administrative databases specialists. The main difficulty regarding the identification of COPD patients from healthcare use is the possible overlap of treatment with other chronic lung diseases. For this reason, we excluded individuals with potential asthma or cystic fibrosis in order to optimize the specificity of the algorithm. Another limitation of this study is the coverage period, which does not extend beyond 2020 due to the study design. Indeed, as patient identification was based on healthcare use in previous years, including a cohort followed in 2021 would have generated a selection bias, as the healthcare system has been heavily impacted by the pandemic in 2020. Finally, causes of death were missing for 8% of deaths, and these were included in the non-COVID-19 deaths, leading to a slight underestimation of COVID-19 deaths in 2020.

In conclusion, this study provides robust findings on the evolution of mortality in COPD patients compared to a control population, reflecting both direct and indirect impacts of the COVID-19 pandemic. The direct impact in terms of COVID-19 mortality was greater in COPD patients than in the control population. However, the indirect impact was unexpectedly favorable, resulting in a substantial decline in deaths from causes other than COVID-19 in the COPD population. This work highlights the contrasting effects of the COVID-19 pandemic on COPD patients, and the specific vulnerability of this population to COVID-19, calling for targeted preventive measures to respond to future respiratory epidemic threats.

## Data Availability

The data analyzed in this study is subject to the following licenses/restrictions: the analysis described in this article was performed in a national database (*Système National des Données de Santé*, SNDS) belonging to a third-party, namely, the French Ministry of Health. Access to this database is available to researchers on reasonable demand, after proper training and upon request to the relevant agency, the *Caisse Nationale d’Assurance Maladie* (CNAM), on condition that the analyses envisaged have been authorized by the French Data Protection Agency (*Commission Nationale de l’Informatique et des Libertés*, CNIL). Requests to access these datasets should be directed to snds.cnam@assurance-maladie.fr.
